# Population Genetic Structure of Marine Leech, *Pterobdella arugamensis* in Indo-West Pacific Region

**DOI:** 10.3390/genes13060956

**Published:** 2022-05-26

**Authors:** Syakirah Azmey, Hussein Taha, Gunanti Mahasri, Muhamad Amin, Ahasan Habib, Min Pau Tan, Takaomi Arai

**Affiliations:** 1 Environmental and Life Sciences Programme, Faculty of Science, Universiti Brunei Darussalam, Jalan Tungku Link, Gadong BE1410, Brunei; hussein.taha@ubd.edu.bn (H.T.); takaomi.arai@ubd.edu.bn (T.A.); 2 Department of Aquaculture, Faculty of Fisheries and Marine, Universitas Airlangga, Kampus C UNAIR, Jl. Mulyorejo Surabaya 60115, Indonesia; gunanti.m@fpk.unair.ac.id (G.M.); muhamad.amin@fpk.unair.ac.id (M.A.); 3 Faculty of Fisheries and Food Science, Universiti Malaysia Terengganu, Kuala Terengganu 21030, Kuala Nerus, Terengganu, Malaysia; a.habib@umt.edu.my; 4 Department of Fisheries and Marine Science, Noakhali Science and Technology University, Noakhali 3814, Bangladesh; 5 Institute Marine Biotechnology, Universiti Malaysia Terengganu, Kuala Terengganu 21030, Kuala Nerus, Terengganu, Malaysia; mptan@umt.edu.my

**Keywords:** hybrid groupers, aquaculture, *Pterobdella arugamensis*, mitochondrial COI, molecular phylogenetic, haplotype network

## Abstract

Grouper aquaculture is rapidly expanding in both tropical and subtropical regions. The presence of marine leeches (*Pterobdella arugamensis*; previously named *Zeylanicobdella arugamensis*) infesting cultured groupers, however, can have a fatal effect on grouper aquaculture production and cause significant economic loss. Understanding the marine leech’s population structure is therefore important to determine its possible distributional origin and distributional mechanisms, which will help monitor and mitigate the infestation. In this study, a total of 84 marine leeches collected from cultured hybrid groupers *Epinephelus* spp. in Brunei Darussalam, Malaysia and Indonesia were identified as *P. arugamensis*, based on morphological and mitochondrial cytochrome c oxidase subunit I gene sequence analyses. These leech samples, together with additional sequences from the GenBank database, were grouped into four genetically distinct haplogroups: (1) Asia Pacific, (2) Borneo, (3) Surabaya and (4) Iran. The four populations were found to be highly diverged from each other. The results also suggested that the samples from the Asia Pacific population could be dispersed and transported from Indonesia.

## 1. Introduction

Groupers (Family *Serranidae*, Subfamily *Epinephelinae*) are commercially valuable marine teleost fish, which are widely distributed in tropical and sub-tropical waters and have become among the most important commodities in the aquaculture sector in many countries, especially in Asia [1]. Grouper culture was initiated in the 1970s in Hong Kong, Malaysia, Singapore, Taiwan and Thailand, before spreading throughout the Southeast Asian region [2,3,4,5,6], and exportation was mainly to China, Japan and Singapore [7]. However, one of the major production constraints in grouper aquaculture is heavy mortality due to diseases [8,9], which has direct impacts on grouper production and economic sustainability of the aquaculture sector of a country [1,6,10]. Viruses, bacteria, fungi, and parasites have been suggested as the causes of the main infectious diseases of cultured groupers [8,11].

A marine ectoparasitic fish leech, *Z. arugamensis* de Silva, 1963, which was first identified in Sri Lanka and belongs to the family Piscicolidae, can infect several teleost fish, including cultured groupers [12,13,14,15,16], by feeding on the blood of its hosts; no other marine leech species has been reported to infest groupers. However, recently, this taxonomic name has been revised to *P. arugamensis* [17]. Any groupers that are infested with this marine leech on their skin will rub their body on any surrounding object, which can cause injuries and ulceration on their skin, leading to secondary infection [18]. In addition, *P. arugamensis* was also reported to be able to transmit haemogregarines and trypanosomes to the fish hosts [19]. Heavy infestation of this marine leech will lead to chronic anaemia [18] and mortality of its fish hosts [12]. *P. arugamensis* is now known to occur around the Indo-West Pacific region, including Brunei Darussalam [16], China [20], India [21], Indonesia [15], Iran [22], Japan [23], Peninsular Malaysia [10,24], Philippines [12], Singapore [25], South Africa [19] and Sri Lanka [26]. However, information on the population structure of *P. arugamensis* is scarce. Research on the population structure of *P. arugamensis* could probably provide an insight into the identification of its distributional origin and its distributional mechanisms, in which this information could lead to the control and mitigation of the leech infestation and prevalence.

Within the Indo-West Pacific region, the marine leech has infected 100% of the cultured hybrid groupers in Brunei Darussalam, and 17 to 83% of the same host from the neighbouring Southeast Asian countries [16]. The cage aquaculture of groupers in Brunei Darussalam began in 1993 but an outbreak of marine leeches has only been observed in farms since 2017 [16]. There is little information available on the origin, cause and mechanism of the outbreak of marine leech in grouper aquaculture in Brunei Darussalam and other parts of the Indo-West Pacific region. In the present study, we collected marine leeches from the Southeast Asian waters, i.e., Brunei Darussalam, Indonesia and Malaysia. Mitochondrial DNA (mtDNA) markers, particularly cytochrome c oxidase subunit 1 (COI), have been proven to be powerful tools for identifying species [27], phylogeographic patterns [28], and the genetic diversity of native and non-native aquatic species [29]. The marine leeches were subjected to identification using both morphological and mitochondrial COI gene analyses. Furthermore, the mtDNA COI gene analysis revealed the population structure of this leech in the Indo-West Pacific region. The results form a basis of discussion on the possible origin and dispersion mechanism of *P. arugamensis* in the Indo-West Pacific region.

## 2. Materials and Methods

**Figure 1:**
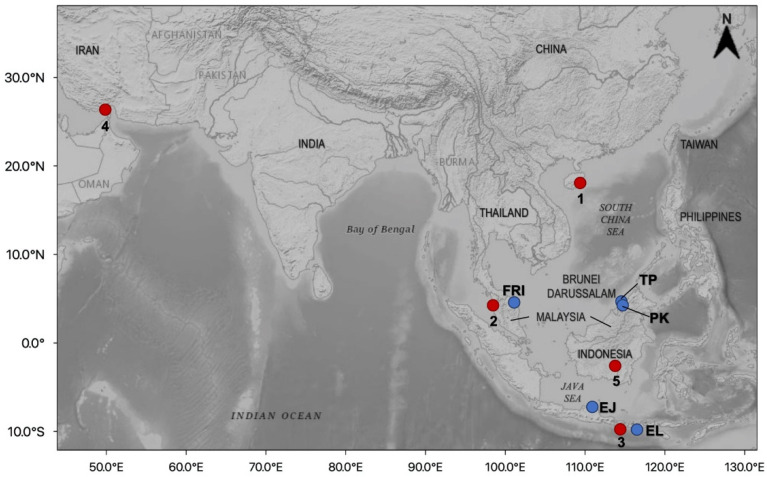
Map showing the collection sites of *P. arugamensis* in Brunei Darussalam, Peninsular Malaysia and Indonesia. Blue circles indicate the sampling locations: FRI: Fisheries Research Institute, TP: Tanjong Pelumpong, PK: Pulau Kaingaran, EJ: Lamongan, East Java, Surabaya and EL: Ekas Bay, East Lombok, West-Nusa Tenggara. Red circles indicate mtDNA COI GenBank sequences: 1. Hainan (China), 2. Penang (Peninsular Malaysia), 3. Bali (Indonesia), 4. Bandar Khamir (Iran), 5. Borneo (specific location is unavailable). Base map is downloaded from the USGS National Map Viewer (open access) at http://viewer.nationalmap.gov/viewer/ (accessed on 14 August 2021).

Marine leech samples and morphological analysis: Marine leeches were collected from cultured hybrid groupers *Epinephelus* sp. (*E. fuscoguttatus ♀* × *E. microdon* ♂; local name “Cantik”) in Brunei Darussalam and Peninsular Malaysia and *Epinephelus* sp. (*E. fuscoguttatus ♀* × *E. lanceolatus* ♂; local name “Cantang”) in Indonesia (Figure 1). In Brunei Darussalam, the marine leeches were collected from two grouper farms at Tanjong Pelumpong (TP) (5°02′01.4″ N, 115°05′52.8″ E) and Pulau Kaingaran (PK) (4°56′59.1″ N, 115°01′36.8″ E). TP is located at the south part of Brunei Bay, facing the South China Sea, and PK is located along the coastline at the mouth of the Brunei River. Samples from both grouper farms were pooled together to form Brunei samples due to their relatively close proximity. In Peninsular Malaysia, the marine leeches were collected at the Fisheries Research Institute (FRI), Besut, Terengganu (5°48′44.1″ N 102°35′20.6″ E). In Indonesia, the marine leeches were collected at Lamongan, East Java, Surabaya (EJ) (6°53′21.5″ S, 112°11′51.7″ E), facing the Java Sea and at Ekas Bay, East Lombok, West-Nusa Tenggara (EL) (8°52′15.5″ S, 116°27′13.5″ E). Our protocols followed the ethical guidelines for the use of animals of the Universiti Brunei Darussalam (UBD) and were approved by the Ethics Committee on Animal Use of the UBD.

For morphological analysis, the samples were immediately stored in in situ water, before being brought back to the laboratory for further analysis. The body length of 75 marine leeches, from the anterior end of the anterior sucker to the posterior end of the posterior sucker, were measured by putting the marine leech in a Petri dish (without any seawater) and measured with a ruler. Morphological identification was then carried out by adapting the method of Ravi and Yahaya [10], in which the marine leeches were treated with lactophenol solution (200 mL lactic acid, 200 g/L phenol, 400 mL glycerol and 200 mL distilled water) for compound microscope observation (Leica DM2500). The external and internal characteristics of the marine leeches observed were then compared to previously documented marine leeches.

Mitochondrial DNA Analysis: Specimens collected from all the sampling locations were preserved in 70% ethanol at room temperature during transportation and at −20 °C for storage. A total of 84 marine leeches were examined for mtDNA COI gene analysis. There were 39 samples from Brunei Darussalam, 16 samples from Surabaya and 18 samples from East Lombok in Indonesia, and 11 samples from Peninsular Malaysia (Table 1). The genomic DNA of ethanol-preserved marine leeches was extracted by using DNeasy Blood and Tissue Kit (QIAGEN, Dusseldorf Germany) according to the manufacturer’s instructions. DNA concentrations were quantified using a Nanodrop 2000 Spectrophotometer. All the extracted DNAs were amplified using polymerase chain reaction (PCR) by targeting the mtDNA COI gene, which were conducted using a forward primer LCO1490 (5′-GGT CAA CAA ATC ATA AAG ATA TTG G-3′) and a reverse primer HCO2198 (5′-TAA ACT TCA GGG TGA CCA AAA AAT CA-3′) [30]. The PCR was conducted in a thermocycler (Cleaver Scientific Ltd., Rugby UK) in a total volume of 50 µL, with the set-up as follows: 25 µL of 2 × Taq PCR Master Mix (QIAGEN, Dusseldorf Germany), 18 µL distilled H_2_O, 2.5 µL of each 10 μM primer and 2 µL of genomic DNA. The PCR conditions were set as follows: initial denaturation for 3 min at 95 °C, followed by 30 cycles of denaturation at the temperature of 95 °C for 30 s, annealing at the temperature of 50 °C for 30 s, extension at the temperature of 72 °C for 1 min, with a final extension at the temperature 72 °C for 5 min. Then, PCR amplicons were purified using a QIAquick Gel Extraction Kit (QIAGEN, Dusseldorf Germany) according to the manufacturer’s instructions and were sent to a service provider for sequencing. Generated sequence trace files were manually edited and assembled using MEGA X [31]. The contig sequences were deposited in the GenBank database with accession numbers MW590405-MW590488, and the sequences were compared for percentage similarity with the sequences in the GenBank database in the National Centre for Biotechnology Information (NCBI, Bethesda, MD, USA) [32] by using BLAST search [33].

The phylogenetic analyses using neighbour-joining (NJ), maximum parsimony (MP) and maximum likelihood (ML) algorithms were performed in MEGA X using default parameters, unless otherwise stated. A total of 11 mtCOI GenBank sequences were included in our analyses. They were from Penang in Peninsular Malaysia (5 sequences) [10], Bali in Indonesia (1 sequence) [15], Hainan in China (1 sequence) [20], Borneo (specific location is not available, 1 sequence) [34] and Bandar Khamir in Iran (3 sequences) [22]. *Pterobdella abditovesiculata* Moore, 1952 (DQ414300; formerly known as *A. abditovesiculata*) was used as an outgroup as it is a closely related species to *P. arugamensis* [17]. ClustalW was used to align the sequences. No internal gaps were observed in the multiple sequence alignment, and the external gaps or missing data were excluded from the analysis, resulting in the analysis of 463 bp (S1 Text). For the NJ tree, the Kimura 2-parameter model was used as it is a widely used model for this algorithm. The MP tree was constructed using the Tree-Bisection-Reconnection (TBR) method with search level 1 and with the initial tree obtained by random addition. The ML tree was constructed after assessment of best-fitting model using MEGA X, resulting in T29 + G (Tamura 3-parameter with a discrete gamma distribution). To construct the ML tree, a heuristic search starting with an initial NJ/BioNJ tree was conducted using the nearest-neighbour-interchange method. All the trees were bootstrapped with 1000 replicates.

MEGA X was also used to measure mean genetic distances within and between groups based on the uncorrected p-distance model. DnaSP version 6 [35] was used to measure DNA polymorphisms at each sampling location and genetic differentiation (fixation index, F_ST_) between groups. DnaSP was also used to generate a haplotype data file in Roehl data format, and this file was then used in Network 10 (www.fluxus-engineering.com, accessed on 11 August 2021) for constructing the haplotype network via the median joining method. A mismatch distribution analysis was carried out using DnaSP. To test if the observed distribution deviated significantly from the distribution expected under the sudden expansion model, the sum of squared deviation (SSD) and Harpending’s raggedness index (r) were computed using Arlequin version 3.5.2.2 [36]. Neutrality tests (Tajima’s D and Fu’s Fs) were also carried out in Arlequin to test for possible deviation from neutrality (equilibrium), which could provide inferences on the demographic history of the samples analysed. The significance level for all tests was *p* < 0.05, except for Fu’s Fs, which was *p* < 0.02. Analysis of Molecular Variance (AMOVA) was carried out using Arlequin.

## 3. Results

### 3.1. Morphological Implications

**Figure 2:**
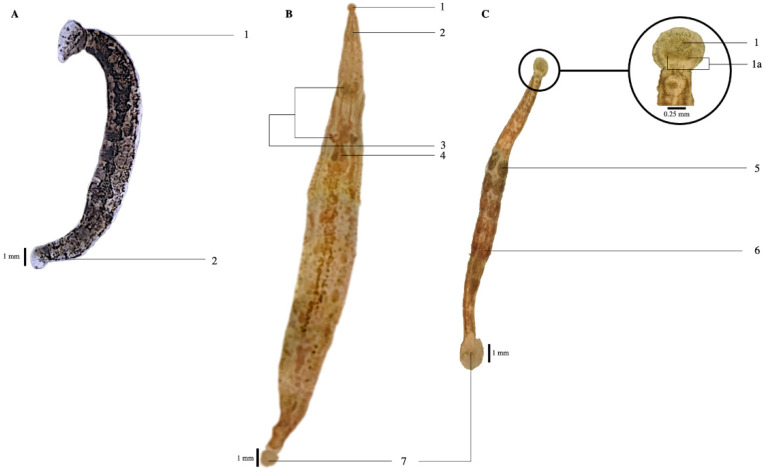
*Pterobdella arugamensis* under light microscopy (**A**–**C**). (**A**) Tanjong Pelumpong, Brunei Darussalam. 1: Anterior sucker, 2: Posterior sucker; (**B**): Pulau Kaingaran, Brunei Darussalam. (**C**): Lombok, Indonesia. (**B**,**C**): The morphology of leech after being treated with lactophenol, 1: Anterior sucker, 1a: a pair of eyes, 2: Proboscis, 3: Mycetomes, 4: Ovisacs, 5: First testisac, 6: Fifth testisac, 7: Posterior sucker.

External morphology: The specimens were soft, elastic and showed cylindrical uniform anatomy, with either black or light brown pigmentation on the adult individuals. The specimens also had a pair of eyespots on their anterior sucker (Figure 2). There were no pulsatile vesicles observed. There were variations in the sizes of the anterior and posterior suckers observed (Figure 2B,C). The total lengths of the adult marine leech overall ranged from 3 to 16 mm. Specifically, the specimens from Peninsular Malaysia (*n* = 11) ranged from 3 to 9 mm, with an average (± standard deviation) of 6 ± 1.69 mm; the specimens from Brunei Darussalam (*n* = 30) ranged from 10 to 15 mm, with an average of 12 ± 1.72 mm, the specimens from Surabaya, Indonesia (*n* = 16) ranged from 10 to 12 mm with an average of 11 ± 0.75 mm, and the specimens from Lombok, Indonesia (*n* = 18) ranged from 12 to 16 mm, with an average of 14 ± 1.19 mm. Internal morphology: Two pairs of mycetomes, ovisacs and five pairs of testisacs could be observed (Figure 2).

Leeches have been the subject of a number of taxonomic revisions and they are now generally accepted under the order Hirudinida [17]. The specimens in this study belong to the suborder Oceanobdelliformes (traditionally accepted as Rhynchobdellida), as they are jawless and have protrusible proboscis. The specimens belong to the family *Piscicolidae* because they infest fish and have a cylindrical body and a defined anterior sucker. The family has over 60 genera, but the specimens have been identified to the genus *Pterobdella* due to the observed two pairs of mycetomes and five pairs of testisacs. The former character is not known in any other genus of piscicolid leeches [17]. There are a few species accepted under this genus, which include *P. abditovesiculata*, *P. amara* Kaburaki, 1921, *P. arugamensis*, *P. leiostomi* Burreson and Thoney, 1991 and *P. platycephali* Ingram, 1957. As the specimens only have one pair of eyespots, they were not identified as *P. abditovesiculata* and *P. leiostomi*, which have more than one pair of eyespots as one of their diagnostic features [37,38]. They were also not identified as *P. platycephali*, which have no eyes and are also larger in size (at least 23 mm in length) [37]. The specimens have pigmentation and indistinct separation of urosome and trachelosome, and, thus, were not identified as *P. amara*, which have distinct separation, lack of pigmentation and elasmobranchs as their hosts. Hence, all the specimens were identified as *P. arugamensis*, which have been reported to have brown to black pigmentation in mature individuals and a total length of up to 15 mm [17]. This species has also been reported to use cultured groupers as its hosts [12,13,14,15,16], which provides further support to the identification. The observed morphological characteristics have also been described by Cruz-Lacierda et al. [12], Murwantoko et al. [15], Nagasawa and Uyeno [23], Chandra [39], Sawyer [40], Burreson [17], Rahayu et al. [41] and Mahasri et al. [42]. 

### 3.2. Genetic Implications

Using BLAST, molecular identification based on mtDNA COI gene confirmed that all the 84 samples were *P. arugamensis*, with 97–100% maximum identity matches with the GenBank sequences of *Z. arugamensis*, including the NCBI reference sequence (GenBank accession no. NC035308 or KY474378 [20]).

Haplotype analysis revealed a total of 16 different haplotypes (H1-H16) from the 84 samples from this study and 11 other samples obtained from the GenBank database (Table 1). DNA polymorphism analysis showed that Surabaya samples were relatively more diverse, with the highest haplotype diversity and nucleotide diversity, and Peninsular Malaysia samples having the lowest haplotype diversity and nucleotide diversity (Table 2).

**Figure 3:**
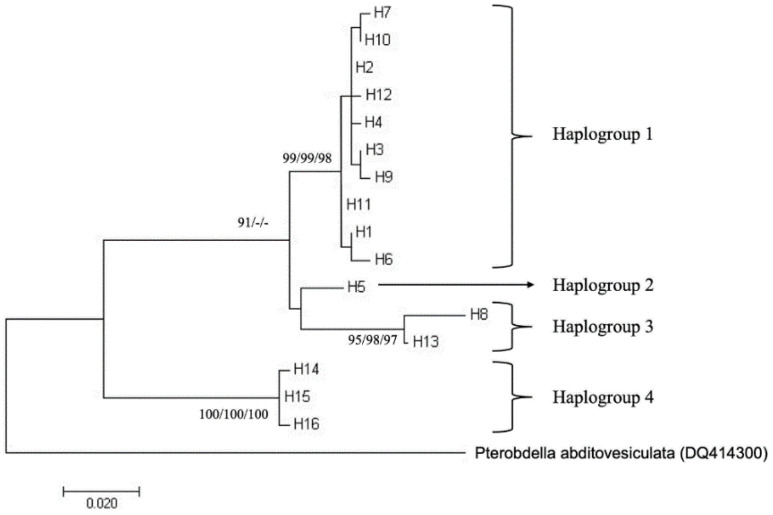
Maximum likelihood tree of 16 haplotypes of *P. arugamensis* based on 463 bp of mtDNA COI gene sequences. *Pterobdella abditovesiculata* was used as an outgroup. The percentages of the bootstrap test (>90%) for maximum likelihood/neighbour joining/maximum parsimony trees are shown next to the branches. The scale bar refers to evolutionary distance and in the unit of number of base substitutions per site.

**Figure 4:**
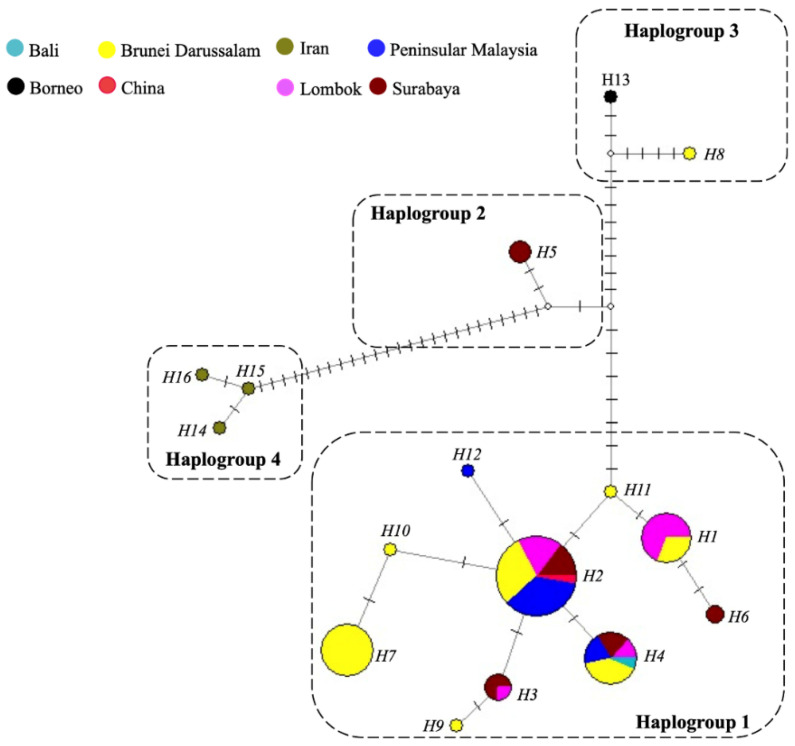
Haplotype network constructed with *P. arugamensis* mtDNA COI sequences. Each colour represents a sample site. The size of the circle is proportional to the number of samples that belong to each haplotype. Each haplotype is labelled as H1 to H16. Each dash, which appears on the line that connects two haplotypes together, symbolises one mutational step. Small white circle refers to median vector which is the hypothesised or missing haplotype.

The population structure of *P. arugamensis* was visualised through phylogenetic trees of the 16 haplotypes and haplotype network. For clustering the haplotypes into different haplogroups, clades with bootstrap values of >90% were examined. Four haplogroups could be clearly observed in all the trees (the ML tree is shown in Figure 3; other trees are in Figure S1), which were well-supported by the bootstrap values. In addition, it should be noted that haplogroup 2 is a sister clade to haplogroup 1 in the NJ and MP trees (with <90% bootstrap values), but in the ML tree, it is a sister clade to haplogroup 3 (with low bootstrap value). Thus, these different haplogroups were considered as distinct groups. These four haplogroups could also be observed in the haplotype network (Figure 4), with the different haplogroups distinctly separated by the higher number of mutational steps between them compared to the lower number of mutational steps between the different haplotypes belonging to the same haplogroup. Ten haplotypes (H1, H2, H3, H4, H6, H7, H9, H10, H11 and H12) are unique to haplogroup 1, one haplotype (H5) is unique to haplogroup 2, two haplotypes (H8 and H13) are unique to haplogroup 3 and three haplotypes (H14, H15 and H16) are unique to haplogroup 4.

Although sample numbers were low in haplogroup 2 (*n* = 3), haplogroup 3 (*n* = 2) and haplogroup 4 (*n* = 3) but not in haplogroup 1 (*n* = 87), genetic differentiation was measured between different haplogroups and it was found that the fixation index (F_ST_) ranged from 0.778 to 0.976 (Table 3). This suggests that the different haplogroups were highly diverged from each other. The mean genetic distances within haplogroups were also measured and ranged from 0.000 to 0.013, suggesting low divergence within each haplogroup. Meanwhile, the mean genetic distances between haplogroups ranged from 0.025 to 0.063 (Table 3), further suggesting that the different haplogroups were highly diverged from each other. AMOVA showed that the variation among the four haplogroups was 91.3%, the variation within the haplogroups was 8.7%, and the F_ST_ value was 0.91 (*p* < 0.05). This further supports that the different haplogroups were highly diverged from each other.

**Figure 5:**
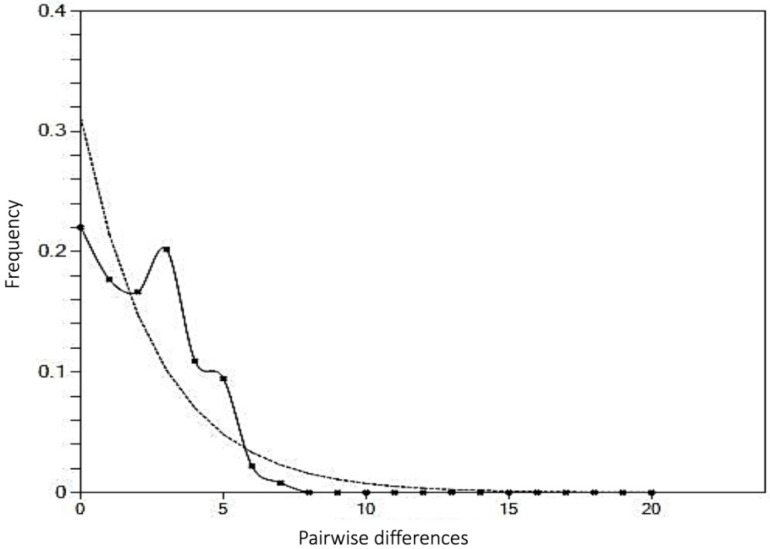
A plot of mismatch distribution. Solid line represents the expected distribution based on the sudden expansion model, whereas the dotted line represents the observed distribution.

A mismatch distribution analysis was carried out based on the observed population structure. However, considering that three of the four haplogroups had low sample numbers, only haplogroup 1 was tested. A plot of the mismatch distribution (Figure 5) shows the observed distribution had a unimodal curve, suggesting a recent population expansion. This was supported by the sum of squared deviation (SSD = 0.004, *p* = 0.83), which did not reject the null hypothesis of a recent demographic expansion. Similarly, Harpending’s raggedness index (r = 0.018, *p* = 0.94) also provided support to population expansion. On the other hand, the neutrality tests did not provide any support to population expansion. Both Tajima’s D and Fu’s Fs values were negative (D = −0.19; Fs = −1.92). Negative values for either test suggest an excess of low frequency polymorphisms, which is a signature for population expansion. However, these values were not significant for Tajima’s D (*p* = 0.42) and Fu’s Fs (*p* = 0.25), which did not indicate significant population expansion.

## 4. Discussion

This study confirmed all marine leeches found on hybrid groupers *Epinephelus* spp. were *P. arugamensis* by means of both morphological and molecular genetic implications. The molecular phylogenetic trees and haplotype network firstly revealed the unique populations structure of *P. arugamensis* in the Indo-West Pacific region (Figure 2 and Figure 3). Four distinct haplogroups were identified, they were (1) Asia Pacific population that consisted of samples from Bali, Lombok and Surabaya in Indonesia, Brunei Darussalam, China and Malaysia (haplogroup 1), (2) Surabaya population that consisted of samples from Surabaya of Indonesia (haplogroup 2), (3) Borneo population that consisted of samples from Brunei Darussalam and Borneo (haplogroup 3), and (4) Iran population that consisted of samples from Iran (haplogroup 4). Interestingly, most of the samples from Brunei Darussalam and Surabaya belonged to the Asia Pacific population, with only one Brunei Darussalam sample belonging to the Borneo population and three Surabaya samples belonging to Surabaya population. This showed that *P. arugamensis* from Brunei Darussalam or Surabaya comprised two genetically distinct populations. This might be due to the introduction of non-native *P. arugamensis* into Brunei Darussalam and Surabaya, which then co-exist with the native population. 

As the hypothesised introduction of the non-native population was relatively a recent event, the effect of gene flow in homogenising both populations through cross fertilisation might not be observed yet. Mitochondrial DNA is of maternal inheritance. One maternal lineage could be more reproductively successful than the other, leading to its fixation although the nuclear DNA could be a mixture of the two populations. In this study, the hypothesised non-native population was sampled more than the hypothesised native population, suggesting that the non-native population might be more reproductively successful. It is also possible that the unique genetic signatures of both the native and non-native populations could persist within the same locality for a long time before one of them is fixed through genetic drift. Furthermore, *P. arugamensis* is a hermaphrodite and is capable of auto fertilisation [24], which would maintain any unique genetic signatures that are present in any populations.

An alternative scenario is that both populations could potentially be non-native, as both might be introduced into the same locality. The outbreak of marine leech in Brunei was first reported in 2017. Prior to 2017, there was no available report of *P. arugamensis* in Brunei, suggesting that the species could be introduced. However, there was a lack of research and interest in Brunei marine leeches to confirm if the species was absent prior to the outbreak. However, if the hypothesised native population was indeed native to Brunei and not introduced, one potential explanation is that this native population was less reproductively successful and, hence, was not able to cause an outbreak that would be noticeable or of particular concern to the grouper farmers.

The Asia Pacific population consisted of genetically homogenous samples from various localities (Bali, Lombok and Surabaya in Indonesia, Brunei Darussalam, China and Malaysia), and this suggests the occurrence of a possible centre of origin for this population. It should be noted that this proposed centre of origin specifically refers to the origin of the haplogroup 1, and not the centre of origin for the species as a whole, which would require further studies and more sampling. Although there is little information known on the dispersal or transportation of this marine leech species, its distribution range is known to be in the Indo-Pacific region, which includes Indonesia, and host species are mostly teleost fishes such as groupers, Mozambique tilapia, red snappers and barramundi [10,12,15,16,19,21,22,23,24,25,26]. Moreover, Indonesia has been the major distributor of grouper fingerlings [1], which are produced from hatcheries in Bali, East Java and Sumatra [43,44]. This suggests that *P. arugamensis* of the Asia Pacific population might originate from somewhere in Indonesia, and it was dispersed or distributed to other localities such as Brunei Darussalam, China and Malaysia through aquaculture industry. The lowest genetic diversity observed in the leech samples from Peninsular Malaysia could perhaps be explained by the introduction of non-native *P. arugamensis* into the site, although this could also be due to the small sample size.

If the leech was expanding to other localities, the population would likely show some genetic signatures of recent population expansion. The mismatch distribution plot and its associated statistical tests supported a recent population expansion. However, the neutrality tests did not support any significant population expansion. A potential explanation for these contradicting results is due to the low number of polymorphic sites observed in the COI gene. This gene is generally more conserved compared to other mitochondrial markers such as cytochrome b and D loop, which makes COI useful for DNA barcoding and makes the other markers a better candidate for mismatch distribution analysis. 

Brunei Darussalam and Malaysia import grouper fingerlings from Indonesia [1,3,5,45,46,47], and *Epinephelus* spp. are generally transported via live fish carriers [1]. This suggests that the distributional routes and mechanisms of *P. arugamensis* from Indonesia to Brunei Darussalam and Malaysia might be through cocoons and/or juveniles of *P. arugamensis* attached on the fingerlings. Furthermore, *P. arugamensis* juveniles are transparent and difficult to find and remove from groupers fingerlings [14]. *P. arugamensis* takes approximately 17 to 22 days to become an adult that can produce on average 11 cocoons within 72 h [14,23,48] and, hence, it would allow further propagation of the marine leech upon arrival to importation destination. The short life cycle and the rapid reproduction together with restricted gene flow due to geographical isolation could contribute to high genetic divergence between populations. Although we did not examine any grouper fingerlings in this study, we could easily observe leeches attaching to the groupers farmed in floating net cages, and when we collected these leeches, we also inadvertently collected their cocoons that hatched into juveniles (Figure S2).

This study is the first to describe that there are four genetically heterogeneous populations of *P. arugamensis* across the Indo-West Pacific region consisting of Asia Pacific, Borneo, Surabaya and Iran populations. The Asia Pacific population is suggested to arise from unintended dispersal through grouper aquaculture. Grouper fingerlings might be the main host for dispersing the marine leech across the countries. Therefore, strict screening for *P. arugamensis* on imported hybrid groupers upon arrival in the country would control and mitigate its prevalence and infestation rate as *P. arugamensis* might be unintentionally dispersed by the host, and once the host has undergone quarantine and thorough check up, there will be a reduction in the spread of marine leeches in the aquaculture system. Furthermore, developing safe biocontrol agents to mitigate the infestation rate of marine leeches using plants might also help the aquaculture system [49,50,51]. 

The samples in this study were barcoded as *P. arugamensis* due to their high similarity (97 to 100% BLAST) with the GenBank reference sequence. Comparison with other closely related species (*P. abditovesiculata*, *P. leiostomi* and *P. amara*) [34] resulted in lower similarity (~90% BLAST). However, the mean genetic distances observed between the different haplogroups ranged from 2.5 to 6.3%. In comparison, the intraspecific mtCOI variation reported in other leeches are generally lower; for example, Annelida (mean 4.89%, median 3.56%), Polychaeta (3.92%, 0.79%), Oligochaeta (6.89%, 0.23%), Hirudinea (2.27%, 0.68%) [52] and Placobdella (mean 1.50%) [53]. This means that the four haplogroups were highly diverged from each other, especially with the haplogroup 4 (Iran population), which brought into question if they are a member of one species or of different species. Leech identification by morphology can be challenging, as some species can be very similar to each other and their taxonomic descriptions are sometimes inadequate [53,54]. A previous study reported that some leech species only differ in their pigmentation, hosts and locality, and speculated that *P. arugamensis* specimens of similar morphological features might be a complex of cryptic species based on its locality (freshwater, estuary and full-strength seawater) [17]. The present study seems to provide molecular evidence of cryptic species. However, this needs to be further investigated and validated. Further study is needed to confirm the identity of the Iran samples, as high genetic distances (~6%) were observed when a comparison was made with this population only.

## 5. Conclusions

This study has firstly revealed the population structure in *P. arugamensis*, in which there are four different haplogroups, including Asia Pacific, Borneo, Surabaya and Iran. The results suggested that the marine ectoparasitic fish leech *P. arugamensis* in the Asia Pacific population could be dispersed and transported from Indonesia. These suggest that strict screening of *P. arugamensis* on imported hybrid groupers upon arrival in the country would be needed to control and mitigate its prevalence and infestation. To understand the dispersion mechanism, further comprehensive studies on the population structure of *P. arugamensis* across the whole distribution range and the use of other DNA markers are required, as *P. arugamensis* is widely distributed in the Indo-West Pacific region.

## Figures and Tables

**Table 1:** Haplotype analysis of *P. arugamensis* and their corresponding geographical location.

Haplotype 1	No. of Samples	Localities (No. of Samples in This Study or GenBank Accession No.)
1	13	Indonesia: Lombok (9 samples) & Brunei Darussalam (4 samples)
2	34	Peninsular Malaysia: Terengganu (10 samples), Indonesia: Lombok (6 samples), Indonesia: Surabaya (5 samples), Brunei Darussalam (10 samples), China: Hainan (KY474378) & Peninsular Malaysia: Penang (KY441720, KY441719)
3	4	Indonesia: Lombok (1 sample) & Indonesia: Surabaya (3 samples)
4	15	Peninsular Malaysia: Terengganu (1 sample), Indonesia: Lombok (2 samples), Indonesia: Surabaya (3 samples), Brunei Darussalam (6 samples), Indonesia: Bali (MH299847) & Peninsular Malaysia: Penang (KY441721, KY441718)
5	3	Indonesia: Surabaya (3 samples)
6	2	Indonesia: Surabaya (2 samples)
7	15	Brunei Darussalam (15 samples)
8	1	Brunei Darussalam (1 sample)
9	1	Brunei Darussalam (1 sample)
10	1	Brunei Darussalam (1 sample)
11	1	Brunei Darussalam (1 sample)
12	1	Peninsular Malaysia: Penang (KY441717)
13	1	Borneo: Unidentified Region (DQ414344)
14	1	Iran (FM208110)
15	1	Iran (FM208111)
16	1	Iran (FM208109)

**Table 2:** DNA polymorphism of *P. arugamensis* from four different localities. Polymorphic sites refer to the sites that show nucleotide differences in a sample of DNA sequences. Haplotypes refer to non-identical DNA sequences. Hd is also known as allele diversity and refers to the probability that two random sequences are different. k refers to the average number of nucleotide differences between any two sequences. π refers to the average number of nucleotide differences per site between any two sequences, and also refers to the probability that two random sequences are different at a given site.

	Lombok	Surabaya	Brunei Darussalam	Peninsular Malaysia
Number of sequences	18	16	39	11
Number of polymorphic sites	7	18	25	1
Number of haplotypes	5	5	9	2
Haplotype diversity, Hd	0.752	0.833	0.773	0.182
Average number of differences, k	2.22	6.27	2.96	0.182
Nucleotide diversity, π	0.00389	0.0110	0.00518	0.000320

**Table 3:** Genetic differentiation (F_ST_) and mean genetic distances of *P. arugamensis*. At the diagonal, within-group genetic distances are shown in bold. Above the diagonal, between-group genetic distances are shown. Minimum and maximum genetic distances are shown in bracket. Below the diagonal, F_ST_ values are shown.

	Haplogroup 1	Haplogroup 2	Haplogroup 3	Haplogroup 4
Haplogroup 1	**0.004 (0.000–0.013)**	0.025 (0.022–0.028)	0.038 (0.032–0.043)	0.063 (0.058–0.067)
Haplogroup 2	0.926	**0.000**	0.030 (0.028–0.032)	0.060 (0.058–0.060)
Haplogroup 3	0.778	0.786	**0.013**	0.061 (0.060–0.063)
Haplogroup 4	0.948	0.976	0.871	**0.003 (0.002–0.004)**

## Data Availability

Data are provided in the article.

## Supplementary Materials

The following supporting information can be downloaded at: https://www.mdpi.com/article/10.3390/genes13060956/s1, Text S1: DNA sequences (463 bp) used. Figure S1: Phylogenetic trees of *P. arugamensis* based on CO1 gene sequences with *P. abditovesiculata* used as an outgroup. (A): Neighbor joining (NJ) tree, (B): Maximum parsimony (MP) tree, (C): Maximum likelihood (ML) tree. Figure S2: Sampling of cultured hybrid groupers, leeches and cocoons from Brunei Darussalam. (A): A scoop net was used to collect cultured hybrid groupers from a floating net cage. (B): A collected grouper *Epinephelus* sp. (*E. fuscoguttatus ♀* × *E. microdon* ♂; local name ‘Cantik’) was immersed temporarily in tap water. Larger adult marine leeches could easily be seen attaching to the fish. The tap water would cause the leeches to detach from the fish. (C): The tap water was filtered to collect the leeches, and cocoons were also inadvertently collected. (D): Collected cocoons (red circles) were placed on a Petri dish, and some of them hatched into juvenile leeches (red arrows).
