# Supplementary material for: Population Genetic Structure of Marine Leech, Pterobdella arugamensis in Indo-West Pacific Region
Source: Genes (Basel). 2022 May 26;13(6):956. doi: 10.3390/genes13060956 (PMC9222843; doi:10.3390/genes13060956)
Supplement: Supplementary file 1 [file genes-13-00956-s001.zip › Figure S1.pdf]

A

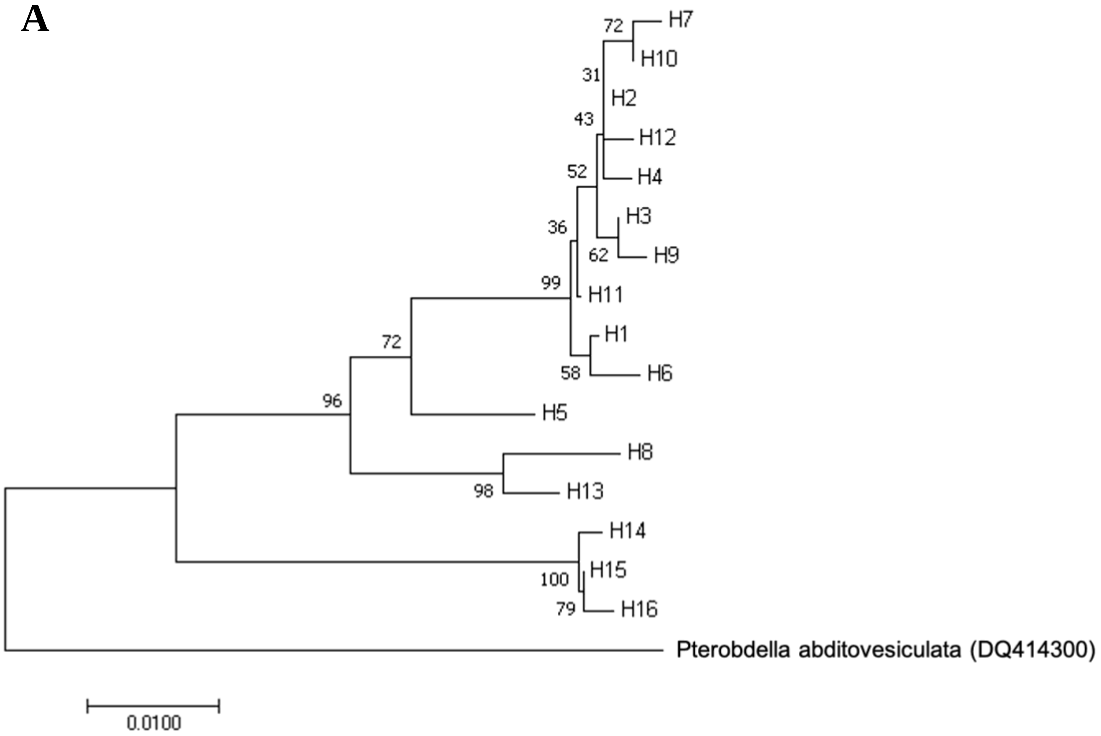
H7
H10
H2
H12
H4
H3
H9
H11
H1
H6
H5
H8
H13
H14
H15
H16
Pterobdella abditovesiculata (DQ414300)
72
31
43
52
36
62
99
72
58
96
98
100
79
0.0100

B

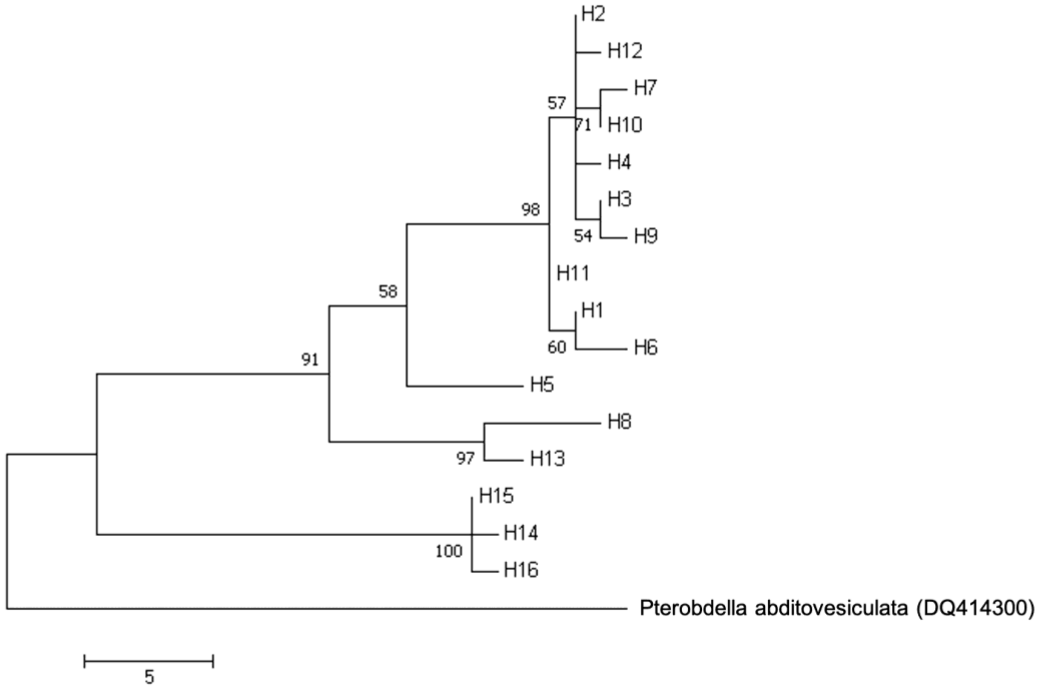
H2
H12
H7
H10
H4
H3
H9
H11
H1
H6
H5
H8
H13
H15
H14
H16
Pterobdella abditovesiculata (DQ414300)
57
71
98
54
58
60
91
97
100
5

C

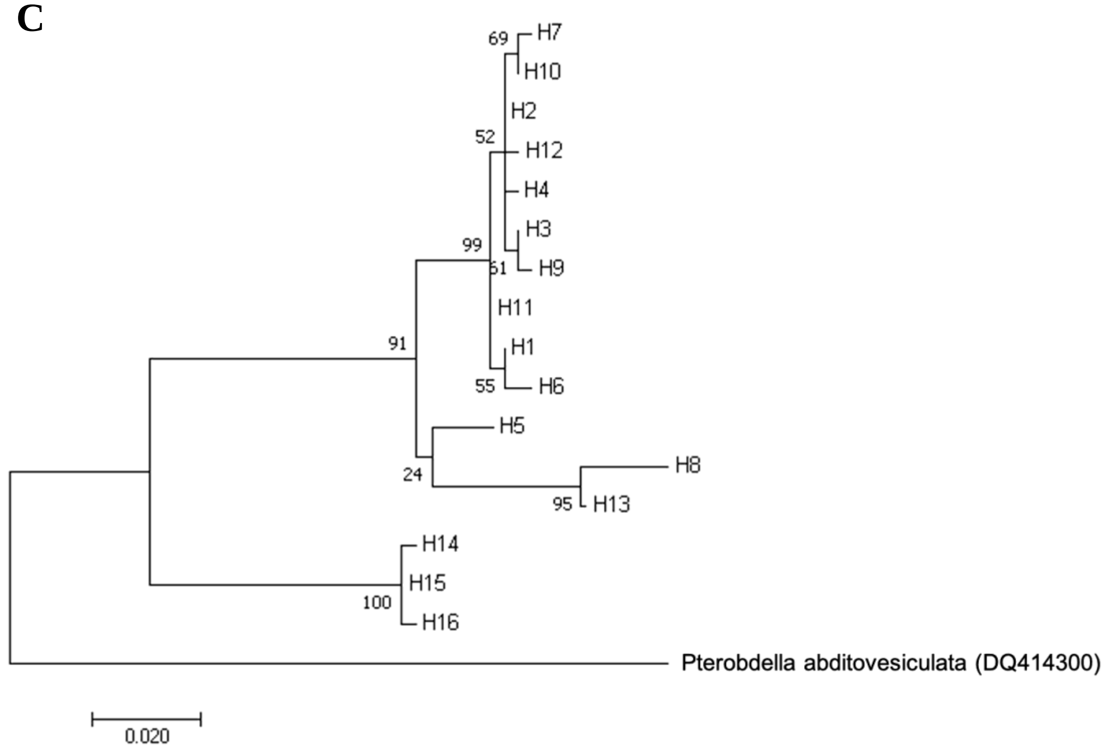

Figure S1. Phylogenetic trees of *P. arugamensis* based on CO1 gene sequences with *P. abditovesiculata* used as an outgroup. A: Neighbor joining (NJ) tree, B: Maximum parsimony (MP) tree, C: Maximum likelihood (ML) tree.
