# Supplementary material for: Population Genetic Structure of Marine Leech, Pterobdella arugamensis in Indo-West Pacific Region
Source: Genes (Basel). 2022 May 26;13(6):956. doi: 10.3390/genes13060956 (PMC9222843; doi:10.3390/genes13060956)
Supplement: Supplementary file 1 [file genes-13-00956-s001.zip › Figure S2.pdf]

A

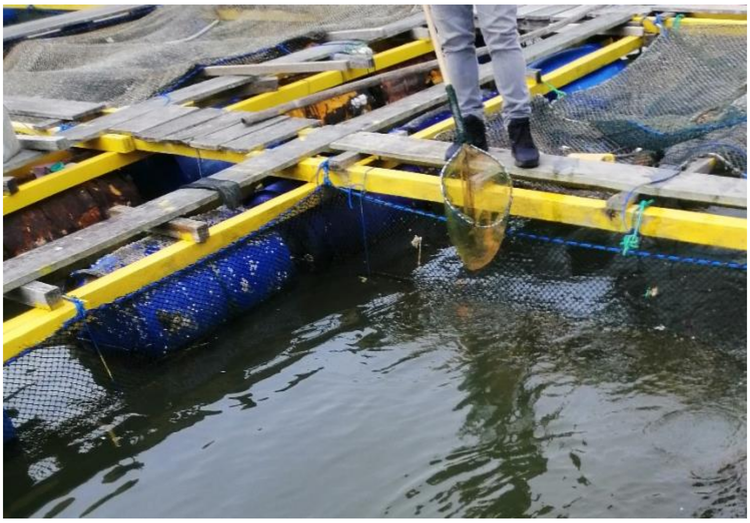

B

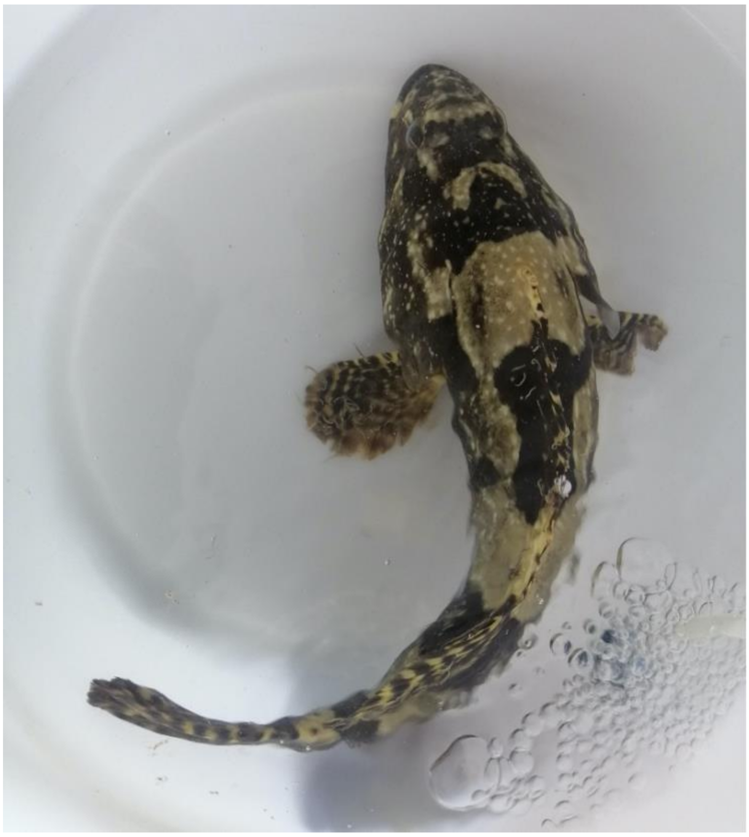

C

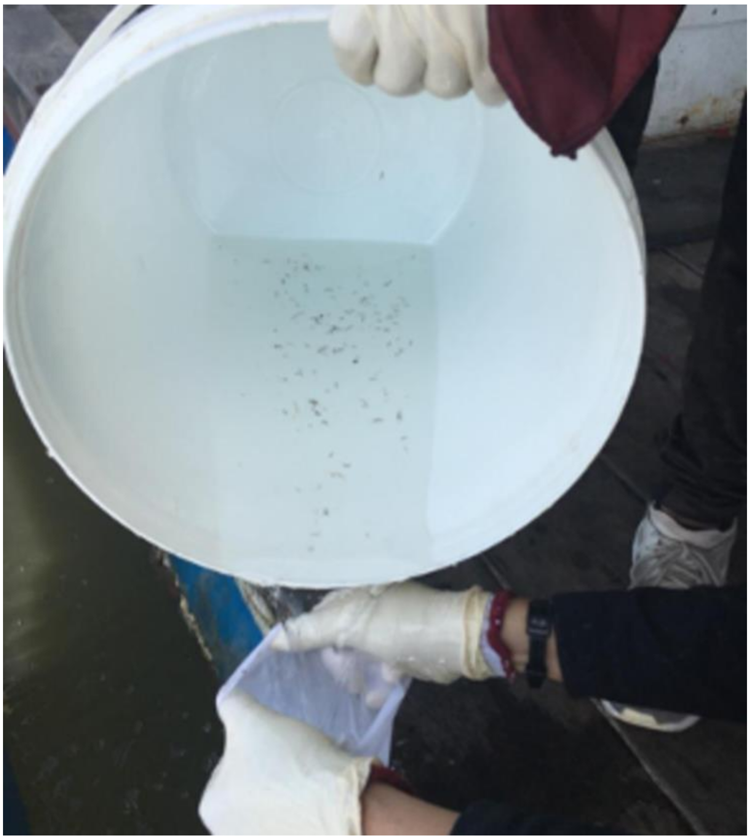

D

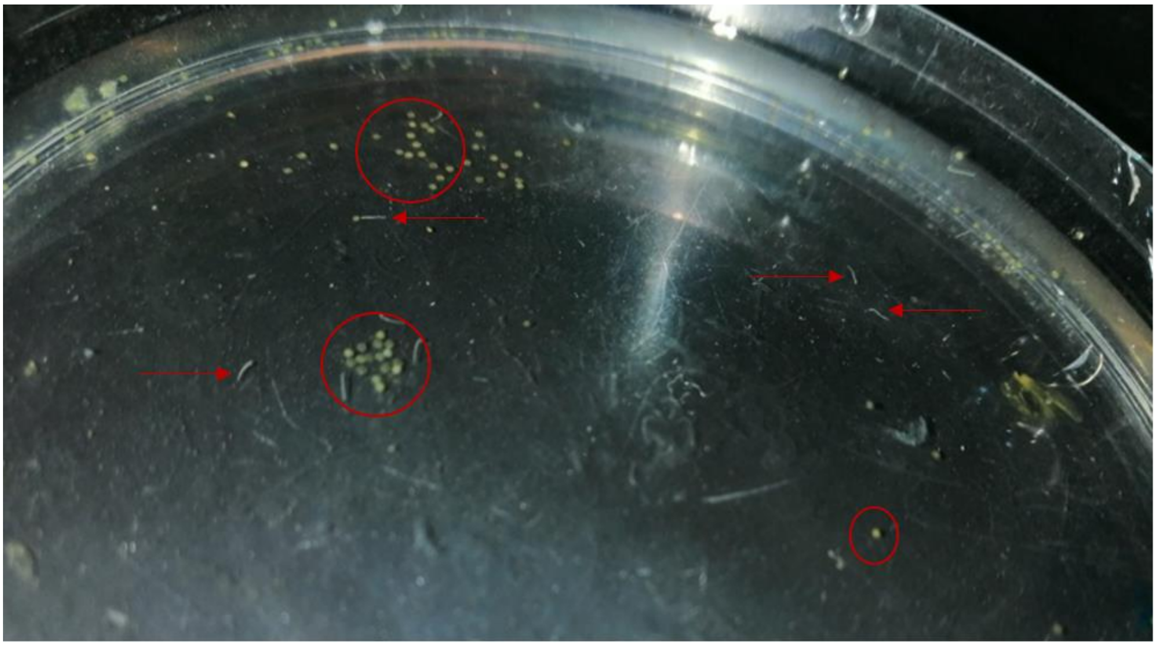

Figure S2. Sampling of cultured hybrid groupers, leeches and cocoons from Brunei Darussalam. A: A scoop net was used to collect cultured hybrid groupers from a floating net cage. B: A collected grouper *Epinephelus* sp. (*E. fuscoguttatus* ♀ × *E. microdon* ♂; local name ‘Cantik’) was immersed temporarily in tap water. Larger adult marine leeches could easily be

seen attaching to the fish. The tap water would cause the leeches to detach from the fish. C: The tap water was filtered to collect the leeches, and cocoons were also inadvertently collected. D: Collected cocoons (red circles) were placed on a Petri dish, and some of them hatched into juvenile leeches (red arrows).
