# Supplementary material for: Population Genetic Structure of Marine Leech, Pterobdella arugamensis in Indo-West Pacific Region
Source: Genes (Basel). 2022 May 26;13(6):956. doi: 10.3390/genes13060956 (PMC9222843; doi:10.3390/genes13060956)
Supplement: Supplementary file 1 [file genes-13-00956-s001.zip › Text S1.pdf]

S1 Text. DNA sequences (463 bp) used.

>H1
AGGTTTGTAATTCGAGCAGAATTATCACAACCAGGTAGATTTATAGAAAATGATCAAATTTATAACTCAATAATTACAGCAC
ATGGGTTAATTATAATTTTTTTTATAGTTATACCAATTCTAATTGGTGGTTTTGGTAACTGGCTAGTACCTTTAATAATTGGTG
CTCCAGACATGGCTTTTCCACGACTTAATAATCTTAGATTTTGATTATTACCACCATCATTAATTCTTCTTTTATCATCTGCATT
AATTGAAAGTGGGGTTGGTACAGGTTGAACAGTCTACCCACCATTATCAGCTAATATAGCTCATTCTGGACCATCAGTAGAT
TTAGCAATTTTTTCTTTACATTTAGCAGGAATTTCATCAATTTTAGGTGCATTAAATTTTATTACAACAATTTTAAATATACGA
TGAAATGGATTAAAATTAGAGCGTATATCATTATTTGTTTGAGCAG

>H2
AGGTTTGTAATTCGAGCAGAATTATCACAACCAGGTAGATTTATAGAAAATGATCAAATTTATAACTCAATAATTACAGCAC
ATGGGTTAATTATAATTTTTTTTATAGTTATACCAATTCTAATTGGTGGTTTTGGTAACTGGCTAGTACCTTTAATAATTGGTG
CTCCAGACATGGCTTTTCCACGACTTAATAATCTTAGATTTTGATTATTACCACCATCATTAATTCTTCTTTTATCATCTGCATT
AATTGAAAGTGGGGTTGGTACAGGTTGAACAGTCTACCCACCATTATCAGCTAATATAGCTCATTCTGGACCATCAGTAGAT
TTAGCAATTTTTTCTTTACATTTAGCAGGAATTTCATCAATTTTAGGTGCATTAAATTTTATTACAACAATTTTAAATATACGA
TGAAATGGGTTAAAATTAGAACGTATATCATTATTTGTTTGAGCAG

>H3
AGGTTTGTAATTCGAGCAGAATTATCACAACCAGGTAGATTTATAGAAAATGATCAAATTTATAACTCAATAATTACAGCAC
ATGGGTTAATTATAATTTTTTTTATAGTTATACCAATTCTAATTGGTGGTTTTGGTAACTGGCTAGTACCTTTAATAATTGGTG
CTCCAGACATGGCTTTTCCACGACTTAATAATCTTAGATTTTGATTATTACCACCATCATTAATTCTTCTTTTATCATCTGCATT
AATTGAAAGTGGGGTTGGTACAGGTTGAACAGTATACCCACCATTATCAGCTAATATAGCTCATTCTGGACCATCAGTAGAT
TTAGCAATTTTTTCTTTACATTTAGCAGGAATTTCATCAATTTTAGGTGCATTAAATTTTATTACAACAATTTTAAATATACGA
TGAAATGGGTTAAAATTAGAACGTATATCATTATTTGTTTGAGCAG

>H4
AGGTTTGTAATTCGAGCAGAATTATCACAACCAGGTAGATTTATAGAAAATGATCAAATTTATAACTCAATAATTACAGCAC
ATGGGTTAATTATAATTTTTTTTATAGTTATACCAATTCTAATTGGTGGTTTTGGTAACTGGCTAGTACCTTTAATAATTGGTG
CTCCAGACATGGCTTTTCCACGACTTAATAATCTTAGATTTTGATTATTACCACCATCATTAATTCTTCTTTTATCATCTGCATT
AATTGAAAGTGGGGTTGGTACAGGTTGAACAGTCTACCCACCATTATCAGCTAATATAGCTCATTCTGGGCCATCAGTAGAT
TTAGCAATTTTTTCTTTACATTTAGCAGGAATTTCATCAATTTTAGGTGCATTAAATTTTATTACAACAATTTTAAATATACGA
TGAAATGGGTTAAAATTAGAACGTATATCATTATTTGTTTGAGCAG

>H5
AGATTTGTAATTCGAGCAGAACTATCACAACCAGGTAGATTTATAGAAAATGATCAAATTTATAATTCAATAATTACGGCAC
ATGGGTTAATTATAATTTTTTTTATAGTTATACCAATTTTAATTGGTGGTTTTGGTAACTGGTTAGTACCTTTAATAATTGGTG
CTCCAGATATGGCTTTCCCACGACTTAATAATCTTAGATTTTGATTATTACCACCATCATTAATTCTTCTTTTATCATCTGCATT
AATTGAAAGTGGGGTTGGTACAGGTTGAACAGTTTACCCACCATTAGCAGCTAATATAGCTCATTCTGGACCATCAGTAGAT
TTAGCAATTTTTTCTTTACATTTAGCAGGAATTTCATCAATTTTAGGTGCATTAAATTTTATTACAACAATTTTAAATATACGA
TGAAATGGATTAAAATTAGAACGTATATCATTATTTGTTTGAGCAG

>H6
AGGTTTGTAATTCGAGCAGAATTATCACAACCAGGTAGATTTATAGAAAATGATCAAATTTATAACTCAATAATTACAGCAC
ATGGGTTAATTATAATTTTTTTTATAGTTATACCAATTCTAATTGGTGGTTTTGGTAACTGGCTAGTACCTTTAATAATTGGTG
CTCCAGACATGGCTTTTCCACGACTTAATAATCTTAGATTTTGATTATTACCACCATCATTAATTCTTCTTTTATCATCTGCATT
AATTGAAAGTGGGGTTGGTACAGGTTGAACAGTATACCCACCATTATCAGCTAATATAGCTCATTCTGGACCATCAGTAGAT
TTAGCAATTTTTTCTTTACATTTAGCAGGAATTTCATCAATTTTAGGTGCATTAAATTTTATCACAACAATTTTAAATATACGA
TGAAATGGATTAAAATTAGAGCGTATATCATTATTTGTTTGAGCAG

>H7
AGGTTTGTAATTCGAGCAGAATTATCACAACCAGGTAGATTTATAGAAAATGATCAAATTTATAACTCAATAATTACAGCAC
ATGGGTTAATTATAATTTTTTTTATAGTTATACCAATTCTAATTGGTGGTTTTGGTAACTGGCTAGTACCTTTAATAATTGGTG
CTCCAGACATGGCTTTTCCACGACTTAATAATCTTAGATTTTGATTATTACCACCATCATTAATTCTTCTTTTATCATCTGCATT
AATTGAAAGTGGGGTTGGTACAGGTTGGACAGTCTACCCACCATTATCAGCTAATATAGCTCATTCTGGACCATCAGTAGAT
TTAGCAATTTTTTCTTTGCATTTAGCAGGAATTTCATCAATTTTAGGTGCATTAAATTTTATTACAACAATTTTAAATATACGA
TGAAATGGGTTAAAATTAGAACGTATATCATTATTTGTTTGAGCAG

>H8
AGGTTTGTAATTCGAGCAGAATTATCACAACCAGGTAGATTTATAGAAAATGATCAAATTTATAATTCAATAATTACAGCAC
ATGGATTAATTATAATTTTTTTTATAGTTATACCAATTTTAATTGGTGGTTTTGGTAACTGATTGGTACCTTTAATAATTGGTG
CTCCAGATATAGCTTTTCCACGACTTAATAATCTTAGATTTTGATTATTACCTCCATCATTAATTCTTCTTTTATCATCTGCATT
AATTGAAAGTGGAGTTGGTACAGGTTGAACAATTTATCCACCATTAGCAGCTAATATAGCCCATTCAGGTCCATCAGTAGAT
TTAGCAATTTTTTCTTTACATTTAGCAGGAATTTCATCAATTTTAGGTGCATTAAATTTTATTACAACAATTTTAAATATACGA
TGAAATGGATTAAAATTAGAACGTATATCATTATTTGTTTGAGCAG

>H9
AGGTTTGTAATTCGAGCAGAATTATCACAACCAGGTAGATTTATAGAAAATGATCAAATTTATAACTCAATAATTACAGCAC
ATGGGTTAATTATAATTTTTTTTATAGTTATACCAATTCTAATTGGTGGTTTTGGTAACTGGCTAGTACCTTTAATAATTGGTG
CTCCAGACATGGCTTTTCCACGACTTAATAATCTTAGATTTTGATTATTACCACCATCATTAATTCTTCTTTTATCATCTGCATT
AATTGAAAGTGGGGTTGGTACAGGTTGAACAGTATACCCACCATTATCAGCTAATATAGCTCATTCTGGACCATCAGTAGAT
TTAGCAATTTTTTCTTTACATTTAGCAGGAATTTCATCAATTTTAGGTACATTAAATTTTATTACAACAATTTTAAATATACGA
TGAAATGGGTTAAAATTAGAACGTATATCATTATTTGTTTGAGCAG

>H10
AGGTTTGTAATTCGAGCAGAATTATCACAACCAGGTAGATTTATAGAAAATGATCAAATTTATAACTCAATAATTACAGCAC
ATGGGTTAATTATAATTTTTTTTATAGTTATACCAATTCTAATTGGTGGTTTTGGTAACTGGCTAGTACCTTTAATAATTGGTG
CTCCAGACATGGCTTTTCCACGACTTAATAATCTTAGATTTTGATTATTACCACCATCATTAATTCTTCTTTTATCATCTGCATT
AATTGAAAGTGGGGTTGGTACAGGTTGAACAGTCTACCCACCATTATCAGCTAATATAGCTCATTCTGGACCATCAGTAGAT
TTAGCAATTTTTTCTTTGCATTTAGCAGGAATTTCATCAATTTTAGGTGCATTAAATTTTATTACAACAATTTTAAATATACGA
TGAAATGGGTTAAAATTAGAACGTATATCATTATTTGTTTGAGCAG

>H11
AGGTTTGTAATTCGAGCAGAATTATCACAACCAGGTAGATTTATAGAAAATGATCAAATTTATAACTCAATAATTACAGCAC
ATGGGTTAATTATAATTTTTTTTATAGTTATACCAATTCTAATTGGTGGTTTTGGTAACTGGCTAGTACCTTTAATAATTGGTG
CTCCAGACATGGCTTTTCCACGACTTAATAATCTTAGATTTTGATTATTACCACCATCATTAATTCTTCTTTTATCATCTGCATT
AATTGAAAGTGGGGTTGGTACAGGTTGAACAGTCTACCCACCATTATCAGCTAATATAGCTCATTCTGGACCATCAGTAGAT
TTAGCAATTTTTTCTTTACATTTAGCAGGAATTTCATCAATTTTAGGTGCATTAAATTTTATTACAACAATTTTAAATATACGA
TGAAATGGATTAAAATTAGAACGTATATCATTATTTGTTTGAGCAG

>H12
AGGTTTGTAATTCGAGCAGAATTATCACAACCAGGTAGATTTATAGAAAATGAACAAATTTATAACTCAATAATTACAGCAC
ATGGGTTAATTATAATTTTTTTTATAGTTATACCAATTCTAATTGGTGGTTTTGGTAACTGGCTAGTACCTTTAATAATTGGTG
CTCCAGACATGGCTTTTCCACGACTTAATAATCTTAGATTTTGATTATTACCACCATCATTAATTCTTCTTTTATCATCTGCATT
AATTGAAAGTGGGGTTGGTACAGGTTGAACAGTCTACCCACCATTATCAGCTAATATAGCTCATTCTGGACCATCAGTAGAT
TTAGCAATTTTTTCTTTACATTTAGCAGGAATTTCATCAATTTTAGGTGCATTAAATTTTATTACAACAATTTTAAATATACGA
TGAAATGGGTTAAAATTAGAACGTATATCATTATTTGTTTGAGCAG

>H13
AGGTTTGTAATTCGAGCAGAATTATCACAACCAGGTAGATTTATAGAAAATGATCAAATTTATAATTCAATAATTACAGCAC
ATGGGTTAATTATAATTTTTTTTATAGTTATACCAATTTTAATTGGTGGTTTTGGTAACTGATTGGTACCTTTAATAATTGGTG
CCCCAGATATAGCTTTCCCACGACTTAATAATCTTAGATTTTGATTATTACCTCCATCATTAATTCTTCTTTTATCATCTGCATT
AATTGAAAGTGGAGTTGGTACAGGTTGAACAGTTTACCCACCATTATCAGCTAATATAGCCCATTCAGGTCCATCAGTAGAT
TTAGCAATTTTTTCTTTACATTTAGCAGGAATTTCATCAATTTTAGGTGCATTAAATTTTATTACAACAATTTTAAATATACGA
TGAAATGGATTAAAATTAGAACGTATATCATTATTTGTTTGAGCAG

>H14
AGATTTGTAATTCGAGCAGAATTATCACAACCAGGTAGATTTATAGAAAATGACCAAATTTATAATTCAATAATTACAGCAC
ATGGTTTAATTATAATTTTTTTTATAGTAATACCTATTTTAATTGGTGGGTTTGGTAATTGATTAGTACCATTAATAATTGGTG
CACCAGACATAGCTTTTCCACGACTTAATAATCTTAGATTTTGATTATTACCACCTTCATTAATTCTTCTTTTATCATCTGCATT
AATTGAAAGTGGTGTAGGTACAGGTTGAACTGTTTATCCGCCATTATCAGCTAATATAGCTCATTCTGGGCCATCAGTGGATT
TAGCAATTTTTTCTTTACATTTAGCGGGAATTTCATCTATTTTAGGTGCATTAAATTTTATTACTACAATTTTAAATATGCGAT
GAAATGGATTGAAATTAGAACGTATATCATTATTTGTTTGAGCAG

>H15
AGATTTGTAATTCGAGCAGAATTATCACAACCAGGTAGATTTATAGAAAATGACCAAATTTATAATTCAATAATTACAGCAC
ATGGTTTAATTATAATTTTTTTTATAGTAATACCTATTTTAATTGGTGGGTTTGGTAATTGATTAGTACCATTAATAATTGGTG
CACCAGACATAGCTTTTCCACGACTTAATAATCTTAGATTTTGATTATTACCACCTTCATTAATTCTTCTTTTATCATCTGCATT
AATTGAAAGTGGTGTAGGTACAGGTTGAACTGTTTATCCGCCATTATCAGCTAATATAGCTCATTCTGGACCATCAGTGGATT
TAGCAATTTTTTCTTTACATTTAGCGGGAATTTCATCTATTTTAGGTGCATTAAATTTTATTACTACAATTTTAAATATGCGAT
GAAATGGATTGAAATTAGAACGTATATCATTATTTGTTTGAGCAG

>H16
AGATTTGTAATTCGAGCAGAATTATCACAACCAGGTAGATTTATAGAAAATGACCAAATTTATAATTCAATAATTACAGCAC
ATGGTTTAATTATAATTTTTTTTATAGTAATACCTATCTTAATTGGTGGGTTTGGTAATTGATTAGTACCATTAATAATTGGTG
CACCAGACATAGCTTTTCCACGACTTAATAATCTTAGATTTTGATTATTACCACCTTCATTAATTCTTCTTTTATCATCTGCATT
AATTGAAAGTGGTGTAGGTACAGGTTGAACTGTTTATCCGCCATTATCAGCTAATATAGCTCATTCTGGACCATCAGTGGATT
TAGCAATTTTTTCTTTACATTTAGCGGGAATTTCATCTATTTTAGGTGCATTAAATTTTATTACTACAATTTTAAATATGCGAT
GAAATGGATTGAAATTAGAACGTATATCATTATTTGTTTGAGCAG

>Aestabdella abditovesiculata (DQ414300)
AGATTTGTTATTCGAGCAGAACTGTCACAACCAGGAAGATTTGTAGAAAATGATCAAATTTATAATTCAATAATTACAGCAC
ATGGCTTAATTATAATTTTCTTTATAGTAATACCAATTTTAATTGGTGGATTTGGTAATTGATTAGTTCCACTAATAATTGGTG
CACCAGACATAGCATTCCCACGACTTAATAATCTTAGATTTTGATTACTACCACCATCACTTATTCTTCTTTTATCATCAGCAT
TAGTTGAAAGGGGGGTAGGTACAGGTTGAACAGTATACCCACCATTATCAGCTAATATAGCCCATTCTGGTCCATCAGTTGA
CCTAGCAATTTTTTCTTTACATTTAGCAGGAATTTCATCTATTTTAGGAGCTTTAAATTTTATTACAACAATTTTAAACATACG
ATGAAAAGGCTTAAAATTAGAGCGTATATCATTATTTGTTTGAGCAG
